# How Do Dental Clinicians Obtain Up-To-Date Patient Medical Histories? Modeling Strengths, Drawbacks, and Proposals for Improvements

**DOI:** 10.3389/fdgth.2022.847080

**Published:** 2022-03-28

**Authors:** Shuning Li, Anushri Singh Rajapuri, Grace Gomez Felix Gomez, Titus Schleyer, Eneida A. Mendonca, Thankam P. Thyvalikakath

**Affiliations:** ^1^Dental Informatics, Indiana University School of Dentistry, Indianapolis, IN, United States; ^2^Center for Biomedical Informatics, Regenstrief Institute, Inc., Indianapolis, IN, United States; ^3^Indiana University School of Medicine, Indianapolis, IN, United States

**Keywords:** medical history, medical consult, electronic dental record, electronic health record, health information exchange, modeling, patient safety

## Abstract

**Background:**

Access to up-to-date patient medical history is essential for dental clinicians (DCs) to avoid potential harm to patients and to improve dental treatment outcomes. The predominant approach for dental clinicians (DCs) to gather patients' medical history is through patient-reported medical histories and medical consults. However, studies reported varied concordance and reliability of patient-reported medical conditions and medication histories compared to the patient medical records and this process also places a significant burden on patients. Information technology tools/platforms such as an integrated electronic health record containing an electronic dental record module may address these issues. However, these integrated systems are expensive and technically complex and may not be easily adopted by DCs in solo and small group practice who provide the most dental care. The recent expansion of regional healthcare information exchange (HIE) provides another approach, but to date, studies on connecting DCs with HIE are very limited. Our study objectives were to model different aspects of the current approaches to identify the strengths and weaknesses, and then model the HIE approach that addresses the weaknesses and retain the strengths of current approaches. The models of current approaches identified the people, resources, organizational aspects, workflow, and areas for improvement; while models of the HIE approach identified system requirements, functions, and processes that may be shared with software developers and other stakeholders for future development.

**Methods:**

There are three phases in this study. In Phase 1, we retrieved peer-reviewed PubMed indexed manuscripts published between January 2013 and November 2020 and extracted modeling related data from selected manuscripts. In Phase 2, we built models for the current approaches by using the Integrated DEFinition Method 0 function modeling method (IDEF0), the Unified Modeling Language (UML) Use Case Diagram, and Business Process Model and Notation (BPMN) methods. In Phase 3, we created three conceptual models for the HIE approach.

**Results:**

From the 47 manuscripts identified, three themes emerged: 1) medical consult process following patient-reported medical history, 2) integrated electronic dental record-electronic health record (EDR-EHR), and 3) HIE. Three models were built for each of the three themes. The use case diagrams described the actions of the dental patients, DCs, medical providers and the use of information systems (EDR-EHR/HIE). The IDEF0 models presented the major functions involved. The BPMN models depicted the detailed steps of the process and showed how the patient's medical history information flowed through different steps. The strengths and weaknesses revealed by the models of the three approaches were also compared.

**Conclusions:**

We successfully modeled the DCs' current approaches of accessing patient medical history and designed an HIE approach that addressed the current approaches' weaknesses as well as leveraged their strengths. Organizational management and end-users can use this information to decide the optimum approach to integrate dental and medical care. The illustrated models are comprehensive and can also be adopted by EHR and EDR vendors to develop a connection between dental systems and HIEs.

## Introduction

The predominant approach for dental clinicians (DCs) to gather patient's medical history is through patient-reported medical histories and medical consults. Studies that evaluated dental patient-reported medical conditions and medication histories reported varied concordance and reliability compared to the patient medical records (1–6), particularly for medical conditions such as diabetes, hypertension, other uncommon cardiovascular disorders and conditions (1), and Sjogren's Syndrome (7). Furthermore, patient-reported medical histories place a significant burden on patients who may have limited medical knowledge, especially older patients with multiple chronic conditions. Studies have reported patient's difficulty recalling their medical and medication histories, which may negatively affect the dental care process (8–10). Therefore, to obtain complete information, the DCs will consult the patients' physicians through medical consults. However, a previous study on medical consults showed a vast difference in the information requested by DCs vs. information returned by medical providers and, in addition, delays in physician office's responses (11). Therefore, it is critical to establish uninterrupted interaction between dental and medical settings to overcome inconsistent documentation and communication problems, and to improve access to up-to-date patient medical history. This interaction could help avoid the potential for fatal accidents and maintain patient safety during dental care (1, 5, 6, 8, 12).

The application of an electronic dental record (EDR) as a module within an electronic health record (EHR) system (hereby referred to as integrated EDR-EHR) is one of the approaches that can address the issues mentioned above and streamline the information gathering process. It can also help bridge different health care settings to achieve a safe, effective, and efficient use of patient-provider interaction time (13). The implementation and use of integrated EDR-EHRs have been growing within large healthcare organizations (HCOs) and federally qualified health centers (FQHCs) where medical and dental practices are co-located and share patients (13–18). However, these systems are expensive and technically complex; it may not be possible for them to be adopted by most DCs who work in independent private dental practices (14, 19, 20). Additionally, these independent, private DCs cannot adopt such EHR-EDR systems without being credentialed to a major HCO. While access to these systems may be possible for this group of DCs sometime in the future (14, 16, 19), connecting the siloed systems without delay to improve patient care is essential, especially for the elderly populations (18, 20, 21).

Since 2009, several federal policies such as the Health Information Technology for Economic and Clinical Health (HITECH) Act and the 21st Century Cures Act has led to regional and statewide development of Health Information Exchange (HIE) platforms with increased interoperability between HER's (22). The financial incentives established by the Centers for Medicare and Medicaid Services (CMS) have increased providers' use of the HIE during clinical encounters (23–25). Therefore, an HIE approach becomes an option for DCs to obtain patient's medical history. This approach could deliver benefits like the integrated EDR-EHR system, enabling access to patient information from multiple HCOs by private and independent DCs. To date, except for one study that reported DC's use of a regional HIE and the type of information accessed, no research exists that even considered the feasibility of connecting DCs with HIE to improve information sharing and communication between dental and medical providers.

Therefore, as the first step to developing an effective HIE approach between medical and dental systems, it is essential to review the existing literature, model the strengths and weaknesses of the current approaches, and identify the HIE approach's critical technical requirements and functions for future development. Modeling has been widely used in the health care industry for process optimization and automation (26, 27). They can diagrammatically represent the interactions between people and resources and the information flow (26, 28–31). Our study objectives were to model different aspects of the current approaches to identify the strengths and weaknesses, and then model the HIE approach that addresses the weaknesses and retain the strengths of current approaches by using the IDEF0 (Integrated DEFinition methods 0) function modeling method, the Unified Modeling Language (UML) use case diagram and Business Process Modeling Notation (BPMN) method. The models of current approaches identified the people, resources, organizational aspects, workflow, and areas for improvement; while models of the HIE approach identified system requirements, functions, and processes that may be shared with HCO management, clinicians, and other stakeholders for future development (for example, software developers and policymakers).

## Materials and Methods

There are three phases in this study. Phase 1 included a scoping review of existing literature to identify the functional and organizational elements involved when DCs obtain patient medical histories through current approaches. These results built the context of the models and the boundary of the system. Phase 2 illustrated the conceptual models of the current approaches, and Phase 3 modeled the HIE approach and presented the strength and weaknesses between the current approaches and the HIE approach.

### Phase 1: Literature Review

The purpose of the literature review is to further expand our understanding of the processes for DCs to obtain patient medical history during a patient's first visit to a dental clinic. The review is a necessary step to ensure that the models built in this study are generalized and can be adopted by others. We chose PubMed as the database to search for literatures, since the review is focused on the clinical processes not the modeling techniques. The search was performed using the following keywords: health information exchange, medical records, health care process modeling, electronic dental record, integrated electronic dental and medical record systems, and medical consults.

Three researchers (AR, GG, and SL) conducted a comprehensive search of PubMed for literatures published between January 2013 and November 2020. Boolean operators such as “OR” and “AND” were used as conjunctions to combine keywords. To be included in this study, the manuscripts had to be English, PubMed indexed, and full length. All researchers first screened the titles and abstracts of each manuscript and identified eligible ones. Only articles focusing on clinical workflows for accessing a patient's medical history were included in the study. Articles on the significance of accessing a patient's medical history and modeling techniques were excluded. Additional manuscripts were also identified through references listed in the eligible ones. Next, the full-length manuscripts were reviewed. The three researchers conducted the reviews independently. The disagreements among researchers were discussed and resolved by consensus. Identified manuscripts were grouped into different themes by the whole research group, including domain experts and informaticians.

The functional and organizational components described in the manuscripts were extracted. Functional aspects involved the activities, objects, and data managed in a process. Organizational aspects described the actors, roles, skills, authorizations over activities, and information management.

### Phase 2: Conceptual Modeling

We modeled the two current approaches for DCs to obtain patient medical information. The processes start from when a patient making an appointment with a dental clinic and ends with either the patient moving on to receiving treatments or being discharged. The illustrated models seek to capture the interdependencies at a system level.

First, supported by our scoping review, we established high-level system requirements by using UML use case diagrams that identify the participant requirements and boundaries of the system. Second, by using the IDEF0 function modeling method, we determined the functions of the processes. Third, the BPMN models depicted the detailed steps and the information flow within the processes. The models represent a top-down approach beginning with a high-level view and then proceed to refine all the details of the most critical functions, increasing the specification toward identifying basic activities (27, 32). The models were reviewed and finalized by the research team that included DCs and informaticians.

#### Use Case Diagrams

Use case modeling has been used to evaluate different health care environments to improve screening, documentation and ease of data access (33). Use case diagrams are referred to as behavior diagrams that are used to describe a set of actions (use cases) that the end-users (actors) can perform with the system or systems (subject) (34). Use case diagrams define the main flow of events, the prerequisites of a processes, the final process of a system even in predefined conditions (35), and how the end-user should interact with the system to perform their services (33, 36–38). They ensured that the correct system was developed by capturing the end-user's point of view (39). Use case diagrams can be used for software development as they are modeled to specify the user interface requirements, the system's functionalities, how the functionalities interact with the users, and its specific requirements (2).

#### IDEF0 Function Models

After specifying the key functions in the use case diagram, we built the IDEF0 function models. IDEF0 models are used to model the decisions, actions, and activities of a system (40), and can help establish the scope of analysis, especially for functional analysis (41). IDEF0 supports a hierarchical structure that can gradually expose details of the system (42). IDEF modeling has been shown to improve communication efficiency among decisions markers and the staff (40). The primary advantage of using IDEF0 is its hieratical and formalism structure leads to the creation of consistent, integrative models (40).

#### BPMN Process Models

BPMN has become the de facto standard for business process diagrams (43). We intend to use the BPMN model to design, manage and realize business processes, and to allow BPMN diagrams to be translated into software process components for the HIE approach (43). The BPMN models can improve the redesign and standardization of processes and allow for partial or complete automation of activities by simplifying processes, reducing the use of resources, and improving the accuracy of the work performed (44). BPMN's notation is independent of any implementation environment (45, 46), which makes it possible to reuse the models in other applications. BPMN is commonly used to model clinical pathways. It can capture the sequence of the activities as well as the actors and the resources organizations can anticipate the behavior of processes, perceive anomalies, inconsistencies, and inefficiencies in their processes (43, 44).

#### Initial Validation of the Models

An initial expert validation was conducted to check the designed conceptual models corresponding to the existing approaches aligning to the needs and the practices of DC's. These domain experts were chosen not only because of their expertise in dental field but also their expertise in health informatics. These experts in the past two decades, have conducted workflow studies in US general dental practices and characterized the dental team's roles, workflow, and tasks during patient examination, and treatment planning (47–49). The goal was to improve the clinical computing environment as well as to design and implement clinical decision support systems (48–50). These studies included observations in clinical settings (47–49), think aloud protocols (51, 52), surveys (53, 54), and focus groups with dental clinicians (55, 56) that identified opportunities for improving the current clinical workflows.

The domain experts met as a group and reviewed the models. Questions arising during the interactions were noted and discussed, and the models were refined based on the group's consensus. Common questions were about the responsibilities on whom has the first point of access to a patient's medical history, and what the actors had to perform in a dental clinic. For example, the researchers had initially considered to use the same set of models for both integrated EDR/EHR approach and HIE approach; but later based on the domain expert's questions and comments, identified differences in data sources and information retrieving methods. This new knowledge resulted in the creating of two separate sets of models for the two approaches. This refinement process was performed iteratively, until there were no further requests from the domain experts and the team achieved a total agreement.

## Results

### Phase 1: Literature Review

**Figure 1:**
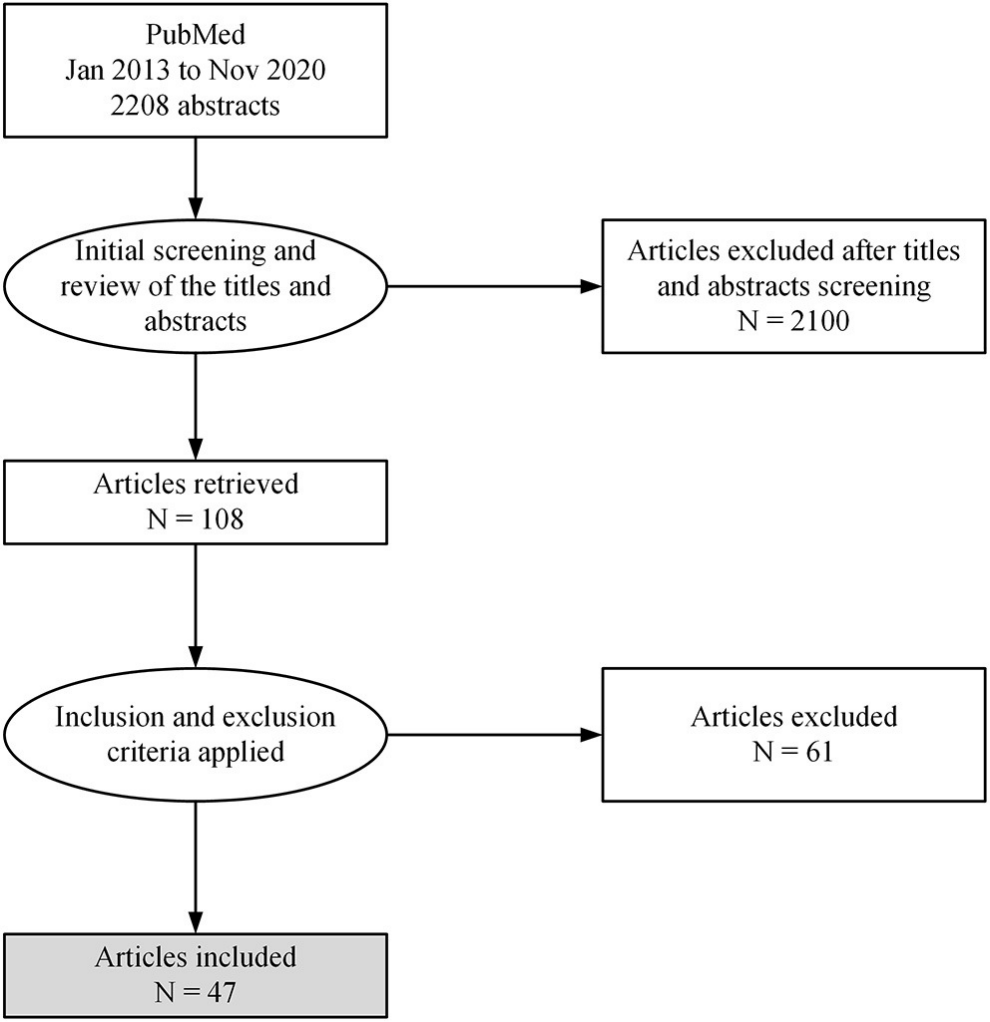
Flowchart representing the process of literature review from the PubMed.

A total of 47 full-length text manuscripts were identified (Figure 1). The manuscripts were divided into three groups based on the DCs approaches to obtaining patient's medical histories (Table 1). The themes were identified as (1) patient-reported medical history followed by medical consults, (2) integrated EDR-EHR, and (3) HIE. The functional and organizational aspects of the process described in each manuscript were extracted. The functional aspects included the list of major functions required by each approach, while the organizational aspects included actors and their responsibilities.

**Table 1:** Functional and organizational aspects extracted from the three themes that emerged from the literature review of dental clinicians obtaining/accessing patient medical histories.

	**# of papers**	**Function**	**Actor (Organizational aspect)**	
			**Name**	**Responsibility**
Medical consult process following patient-reported medical history (1, 4, 5, 18, 39, 57–65)	14	Initiate medical consult	Dental clinician	Send out medical consult requests.
		Resend medical consult	Dental clinician	If the requested information was not received on time, send another request to the same medical provider or a different one.
		Respond to medical consult	Medical provider	Respond to dental providers' information requests.
Integrated electronic dental record-electronic health record (EDR-EHR) system (8, 10, 13, 15, 24, 59, 66–78)	19	Retrieve patient medical history	Dental clinician	Query the EDR/EHR database for patient medical history.
			EDR-EHR platform	Provide patient medical history.
		Initiate medical consult	Dental clinician	If additional information, clearance, and interpretations are needed, create medical consult requests.
			EDR-EHR platform	Send out medical consult requests through a centralized referral module.
Health Information Exchange (HIE) (6, 21, 25, 70, 79–88)	14	Retrieve patient medical history	Dental clinician	Query the HIE database for patient medical history.
			HIE	Provide patient medical history.
		Initiate medical consult	Dental clinician	If additional information, clearance, and interpretations are needed, send out medical consult requests.

### Phase 2: Conceptual Modeling

#### Use Case Diagrams

**Figure 2:**
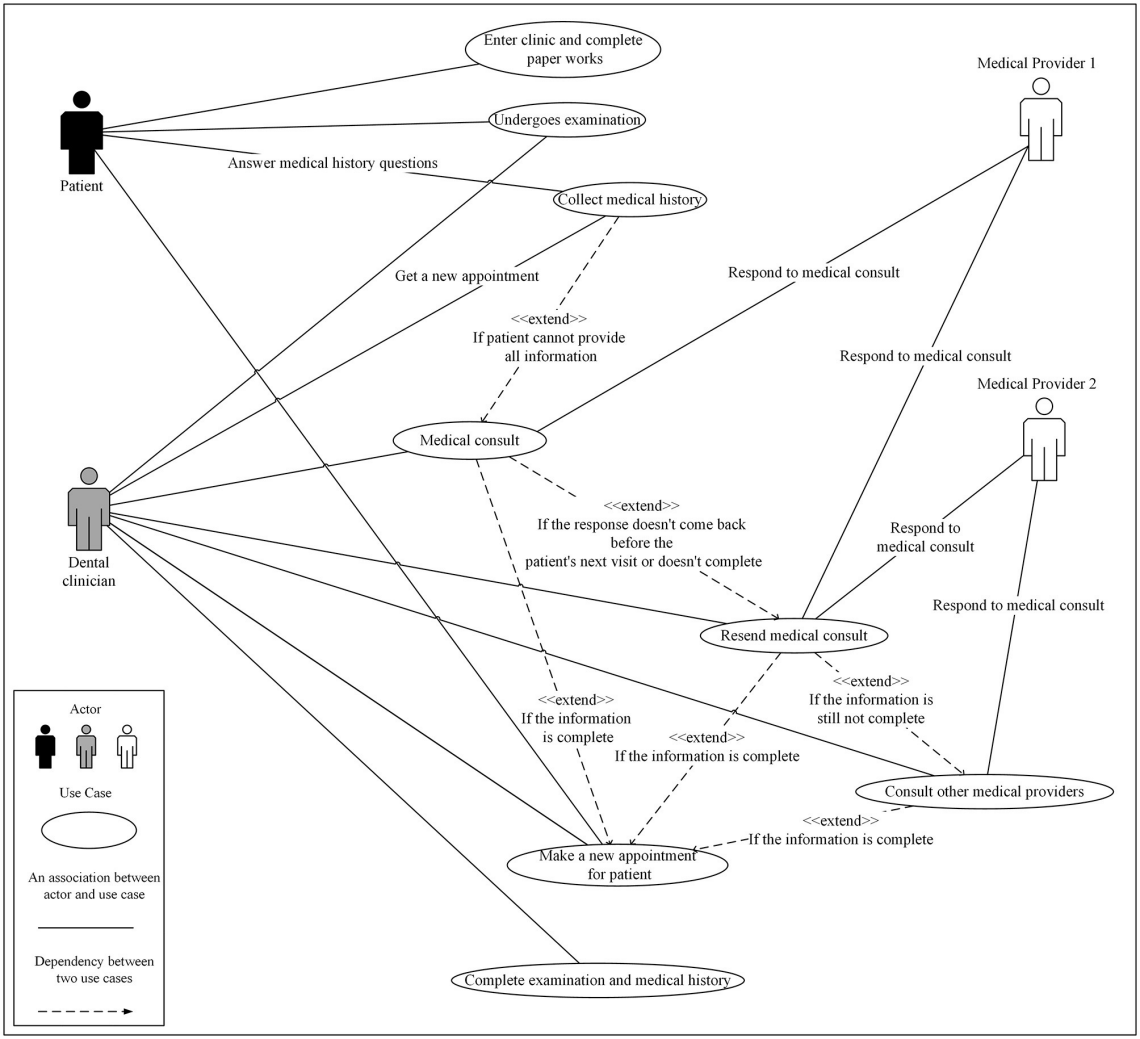
Use case diagram for patient-reported medical history with optional medical consults approach in a dental clinic setting that uses an electronic dental record system and/or is not collocated with a medical clinic setting.

The patient-reported medical history following by medical consults approach had the following actors: the patient, the DCs, and medical providers (Figure 2). The patient was involved in four activities: enter the clinic and complete paperwork, receive an intraoral and extraoral examination by the dental clinician, report medical history and provide primary care physicians and other medical provider's contact information, and schedule a follow-up dental appointment.

In the last three activities, patients worked with DCs to answer questions, provide patient-reported medical history, and schedule appointments. The DCs were involved in six activities. In these use cases, they examined the patient, interviewed the patient, reviewed the patient-reported medical history, initiated medical consults, scheduled further appointments, and completed the examination and medical history. This actor represents not a single person or role but a group of people, including dentists, hygienists, and dental assistants. In addition, administrative staff in a clinic are also consider as part of the patient care team especially in medical history gathering process. The medical providers involved in the three activities related to medical consults. We only used two providers in the model to simulate the possible scenarios, such as notifying the medical provider during medical consults. When DCs do not receive the requested information on time, they would resend the request to the same medical provider or contact a different provider such as a specialist or a healthcare organization. This process would be repeated until the requested information was received.

**Figure 3:**
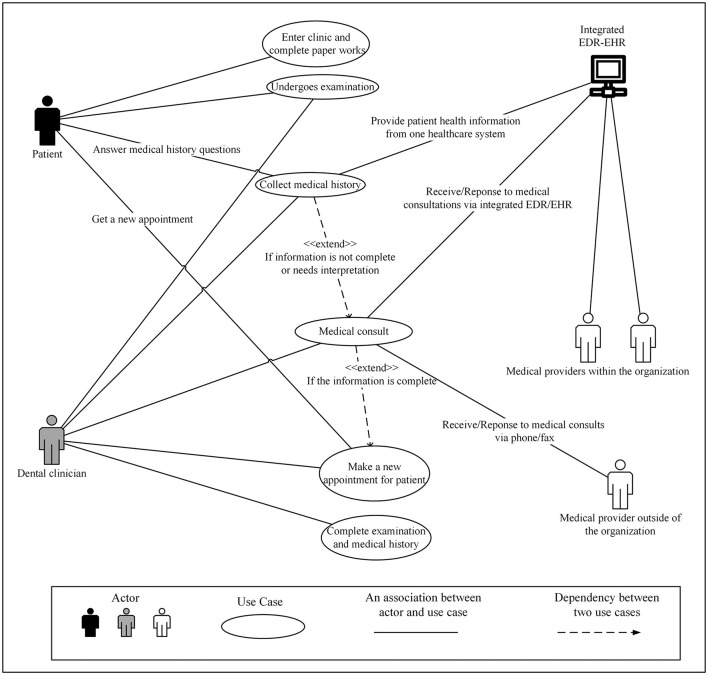
Use Case Diagram for integrated electronic dental record-electronic health record (integrated EDR-EHR) approach as seen in a major healthcare organization (HCO) or federally qualified health center where dental and medical clinics are collocated or affiliated with the same HCO.

There is one more actor, the EDR-EHR system, in the integrated EDR-EHR approach model (Figure 3). Integrated EDR-EHR system is the data source for several use cases. It provides patient medical histories to DCs in the Collect Medical History use case while providing medical providers' contact information in the Medical Consult use case. With the help of the EDR-EHR systems, DCs may only need to reach out to medical providers in complex or rare situations. When they request medical consults, they can ask more specific questions and contact the correct medical providers to avoid repeated consults.

#### IDEF0 Function Models

**Figure 4:**
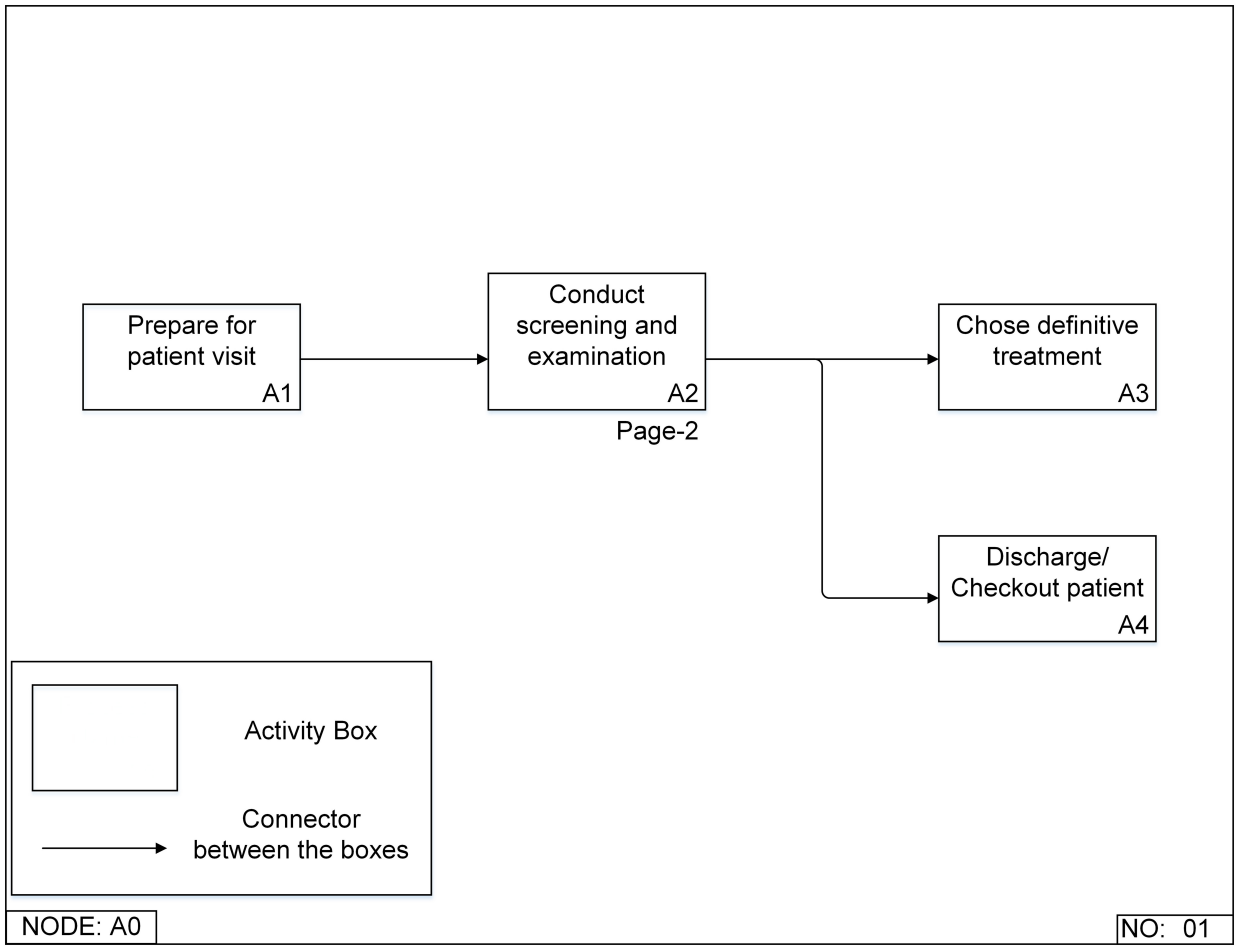
Top-level Integrated DEFinition Method Function (IDEF0) model for a patient's first visit to a dental clinic.

**Figure 5:**
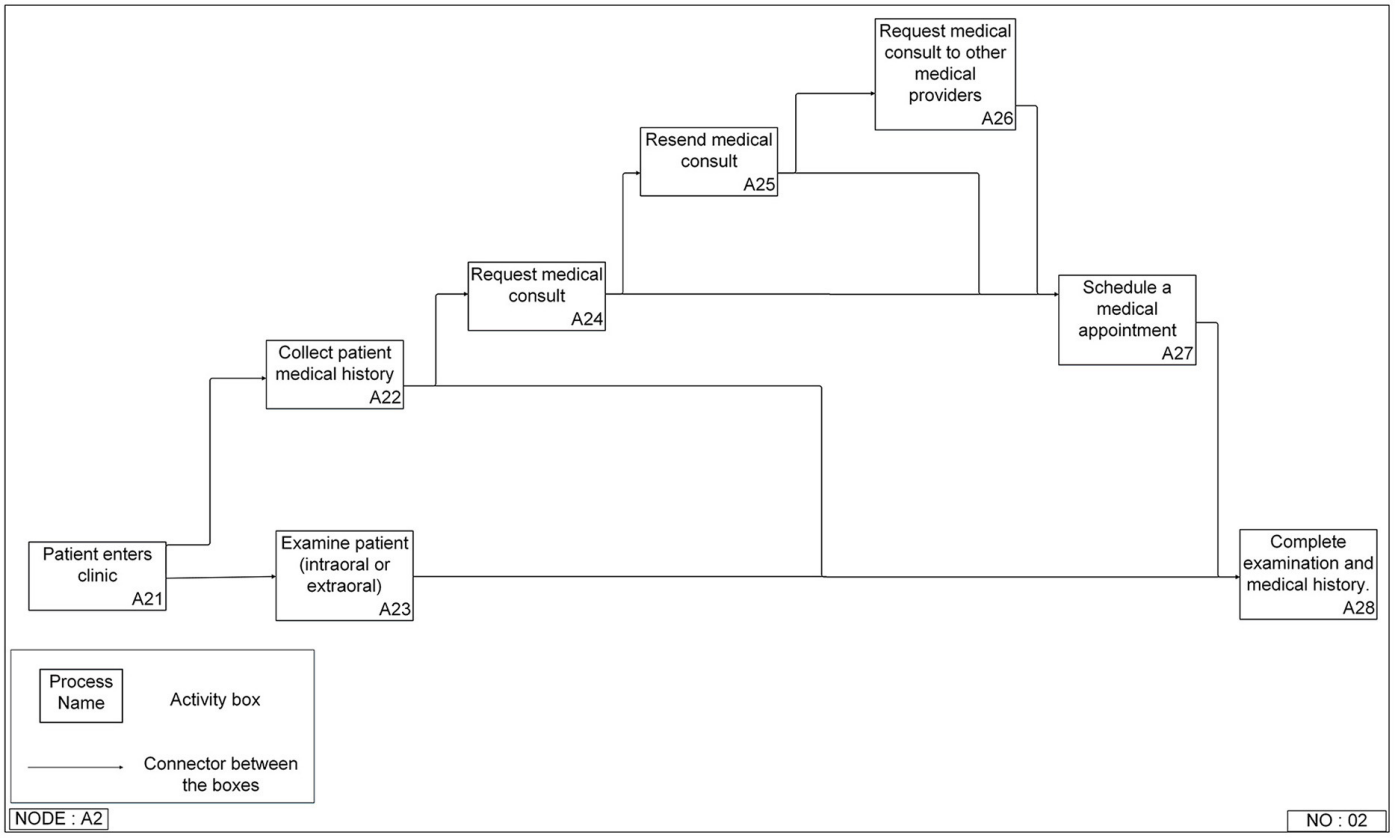
Integrated DEFinition Method Function (IDEF0) model for patient-reported medical history with optional medical consults approach in a dental clinic setting that is not co-located with a medical clinic setting or uses an electronic dental record system.

**Figure 6:**
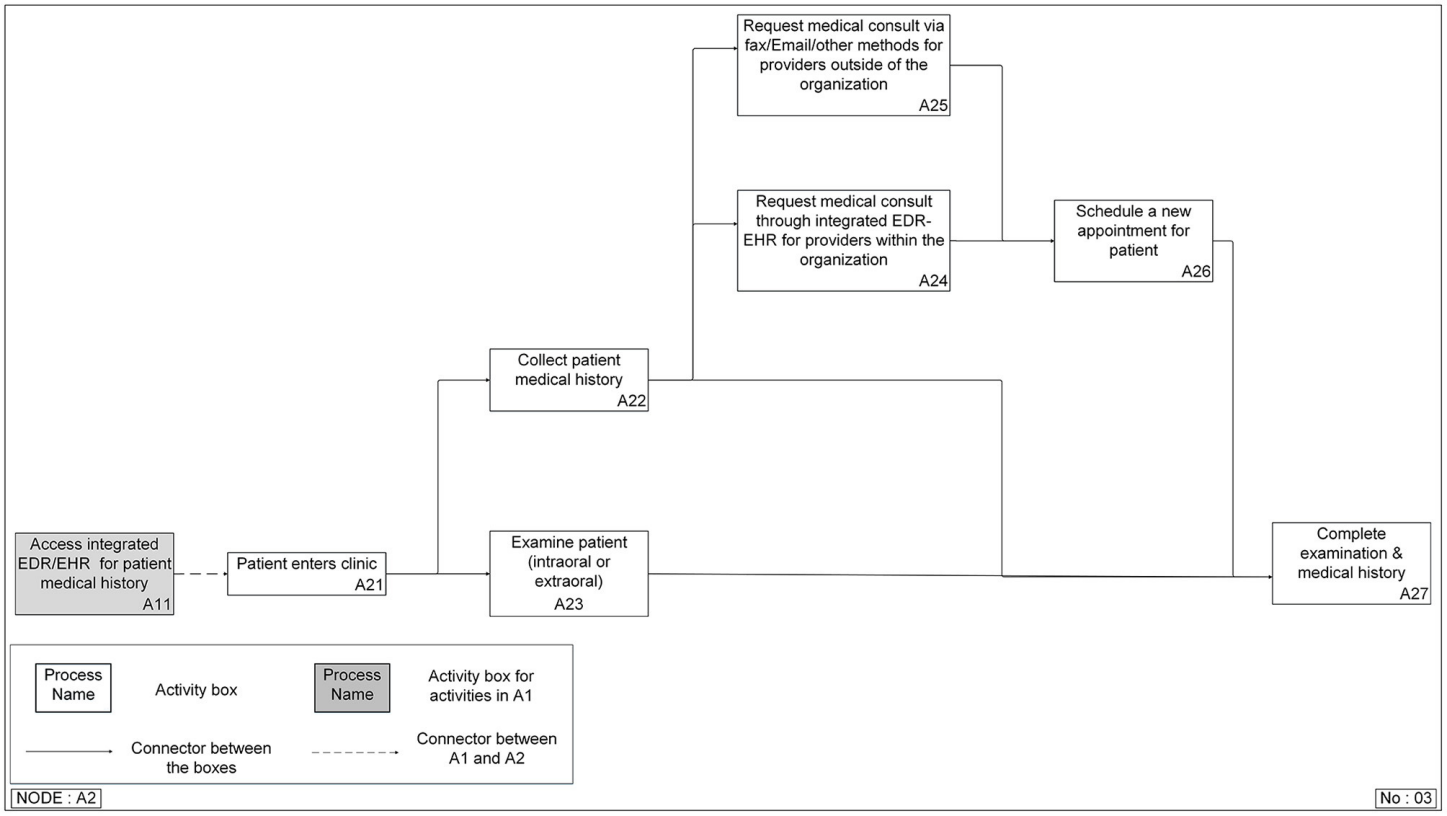
Integrated DEFinition Method Function (IDEF0) model for the integrated electronic dental record-electronic health record (integrated EDR-EHR) approach.

Two levels of the function models were created for all approaches. The top-level model (Figure 4) contained four functions: prepare for a patient visit (A1), conduct screening and examination (A2), choose definitive treatment (A3), and discharge/checkout patients (A4). A3 and A4 are the two possible functions after A2, but only one will be needed for a specific patient. If the DC identified any problem after the screening and examination function (A2), the process would go to choose definitive treatment (A3). Otherwise, the patient would be discharged (A3). This is the same top-level model for all approaches. Accessing patient medical history mainly happened in the conduct screening and examination function (A2), so more detailed breakdown models of this function were built for different approaches (Figures 5, 6).

The patient reported medical history following by medical consult approach model (Figure 5) had eight functions. It started with the patient entering the clinic (A21). Then the process split into two branches: collect patient medical history (A22) and examine patient (A23), both intraoral and extraoral. If all the patient's medical records are clear, it will lead to a complete examination and medical history (A28). However, if there is missing information or the DC has concerns or questions about some of the patient's reported medical history, they will initiate a medical consult request (A24). For instance, the clinician cannot get all the needed information due to delay or non-responses from medical providers. In that case, they will either resend the request to the same medical provider (A25) or a different medical provider (A26). After the medical history is completed, the clinician may need to schedule a new appointment with the patient (A27) to complete the examination and medical history (A28).

The shaded block (A11) in the integrated EDR-EHR model (Figure 6) belongs to A1 (Prepare for the patient visit), the function in the top-level model (Figure 4), which occurs before the patient visits the clinic. However, we included A11 in the second function breakdown model to provide a complete view of the information gathering process. This step reflects one of the significant differences between the two approaches. With the help of information technologies, the clinician can access the patient's medical history before the patient's visit. They can save precious patient-facing time for reviewing and confirming medical conditions with patients, examining patients, and choosing treatment plans.

#### BPMN Process Models

**Figure 7:**
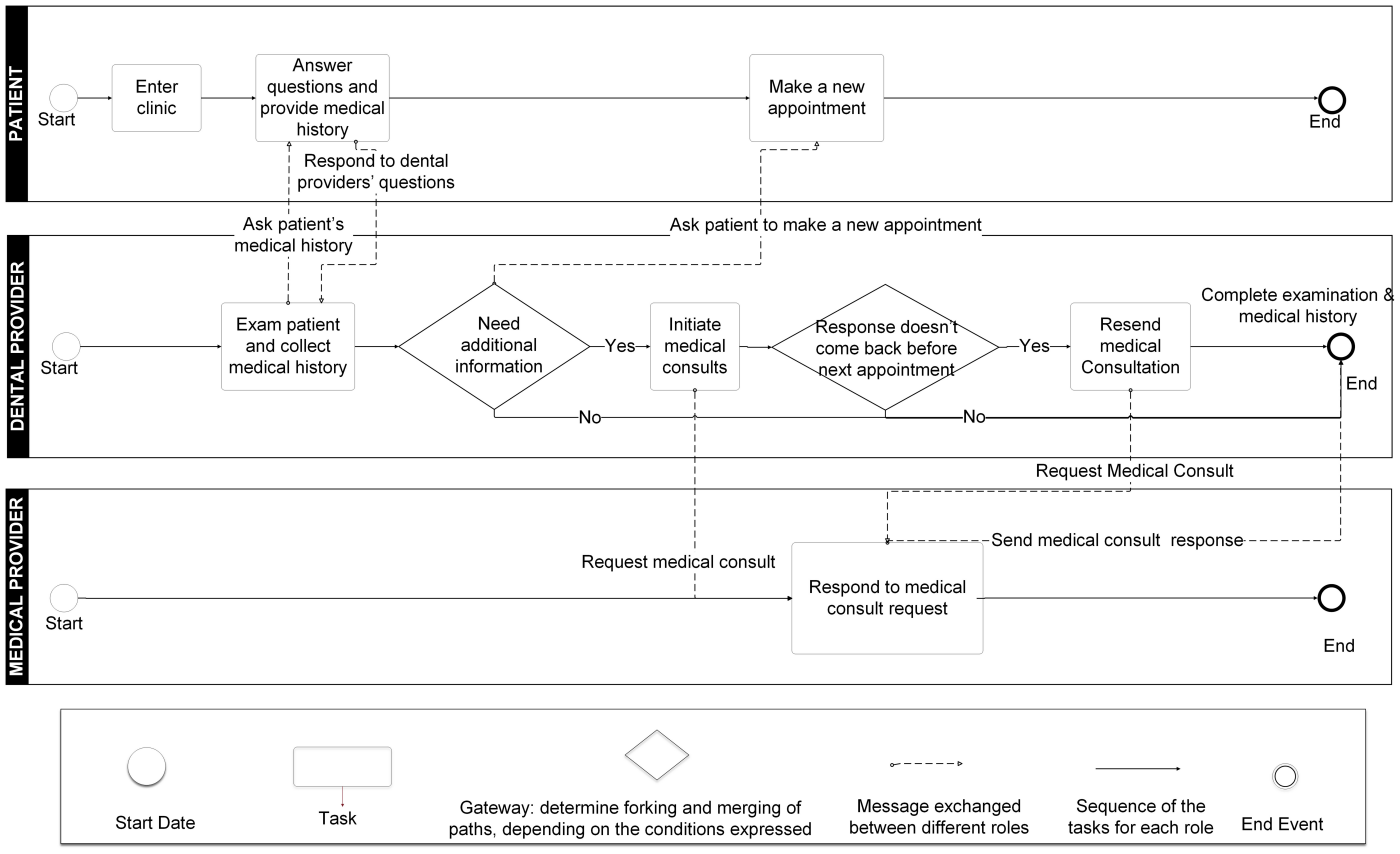
Business Process Modeling Notation (BPMN) model for patient-reported medical history with optional medical consults approach.

The BPMN process models were built based on the UML use case diagrams and the IDEF0 function models. There are three roles in the models (the patient, the dental providers, and the medical providers) in the patient reported medical history following by medical consults approach (Figure 7). We modeled the significant tasks (rectangles) for each role and the communications between different roles.

**Figure 8:**
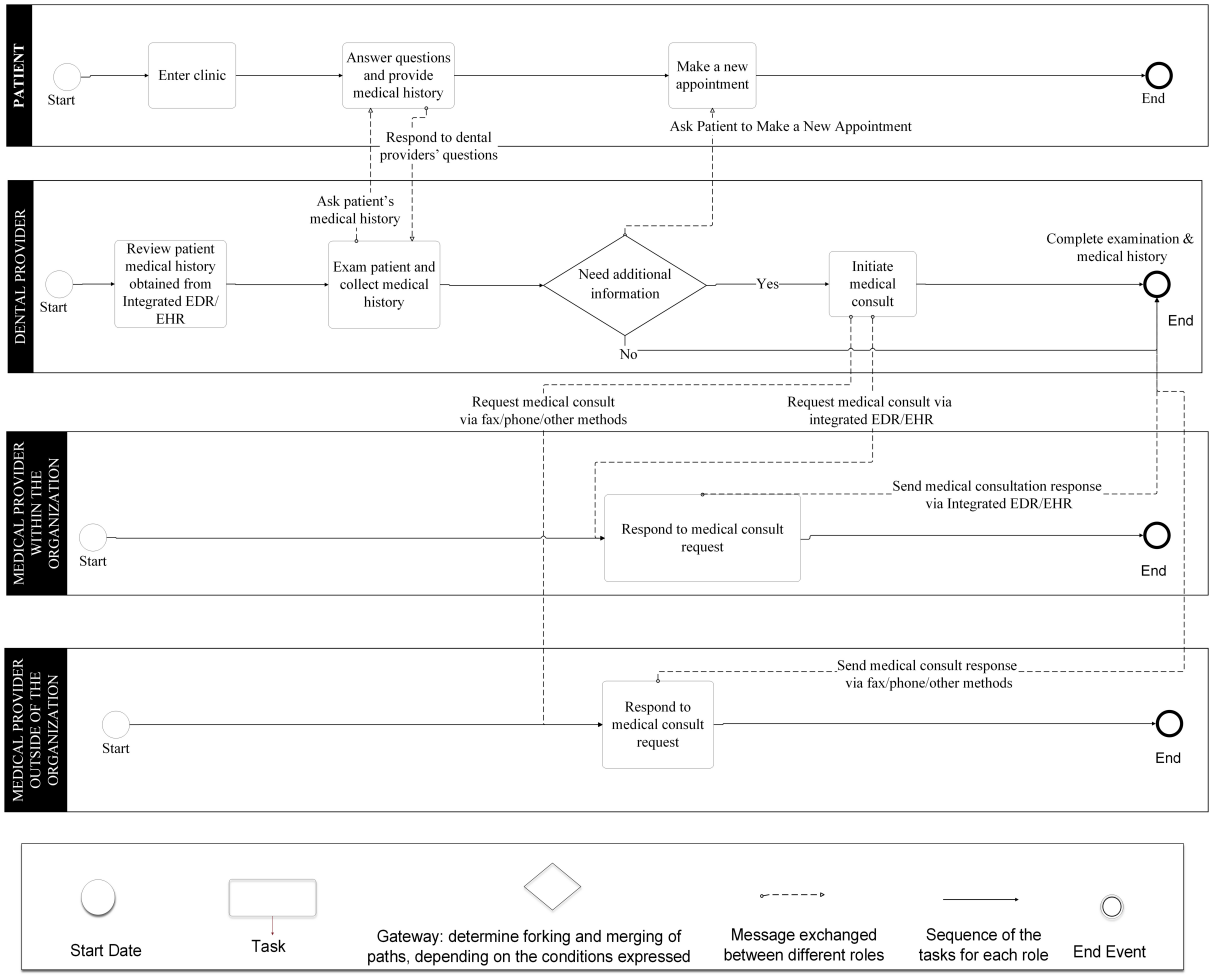
Business Process Modeling Notation (BPMN) model for the integrated electronic dental record-electronic health record (integrated EDR-EHR) approach.

The integrated EDR-EHR approach model (Figure 8) has two separate workflows, one for medical providers within the same HCO as the dental provider and the other one for medical providers outside of the HCO. For medical providers in the same HCO, medical consults can be done by using the integrated EDR-EHR system. While for medical providers who are not in the same HCO as the DC, the medical consults still need to use phone calls, faxes, or emails. For most of the independent DCs who do not belong to any large HCO, medical consults need to follow outside of the organization's path and will not be able to take advantage of the integrated EDR-EHR system.

### Phase 3: The HIE Approach

**Figure 9:**
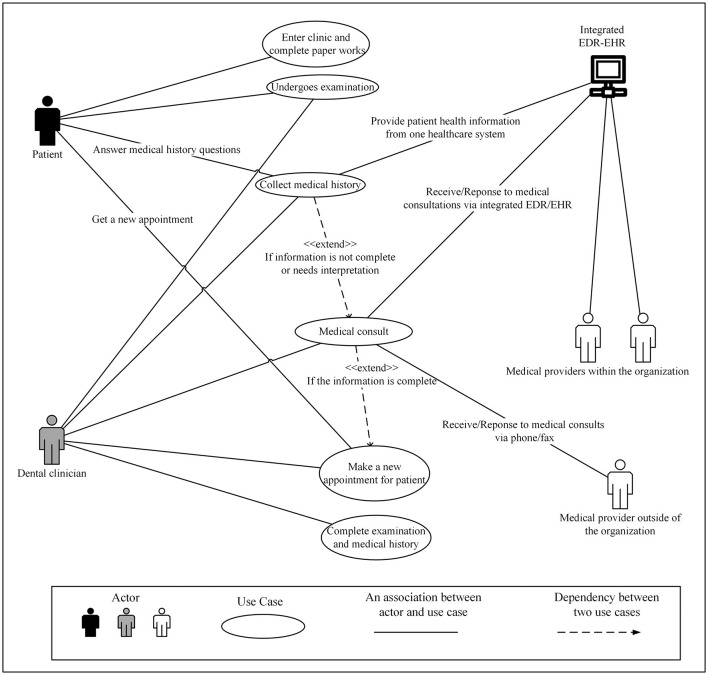
Use Case Diagram for the health information exchange (HIE) approach that can connect standalone dental practices not affiliated with major healthcare organizations with community or regional HIEs to access their patient's medical history.

**Figure 10:**
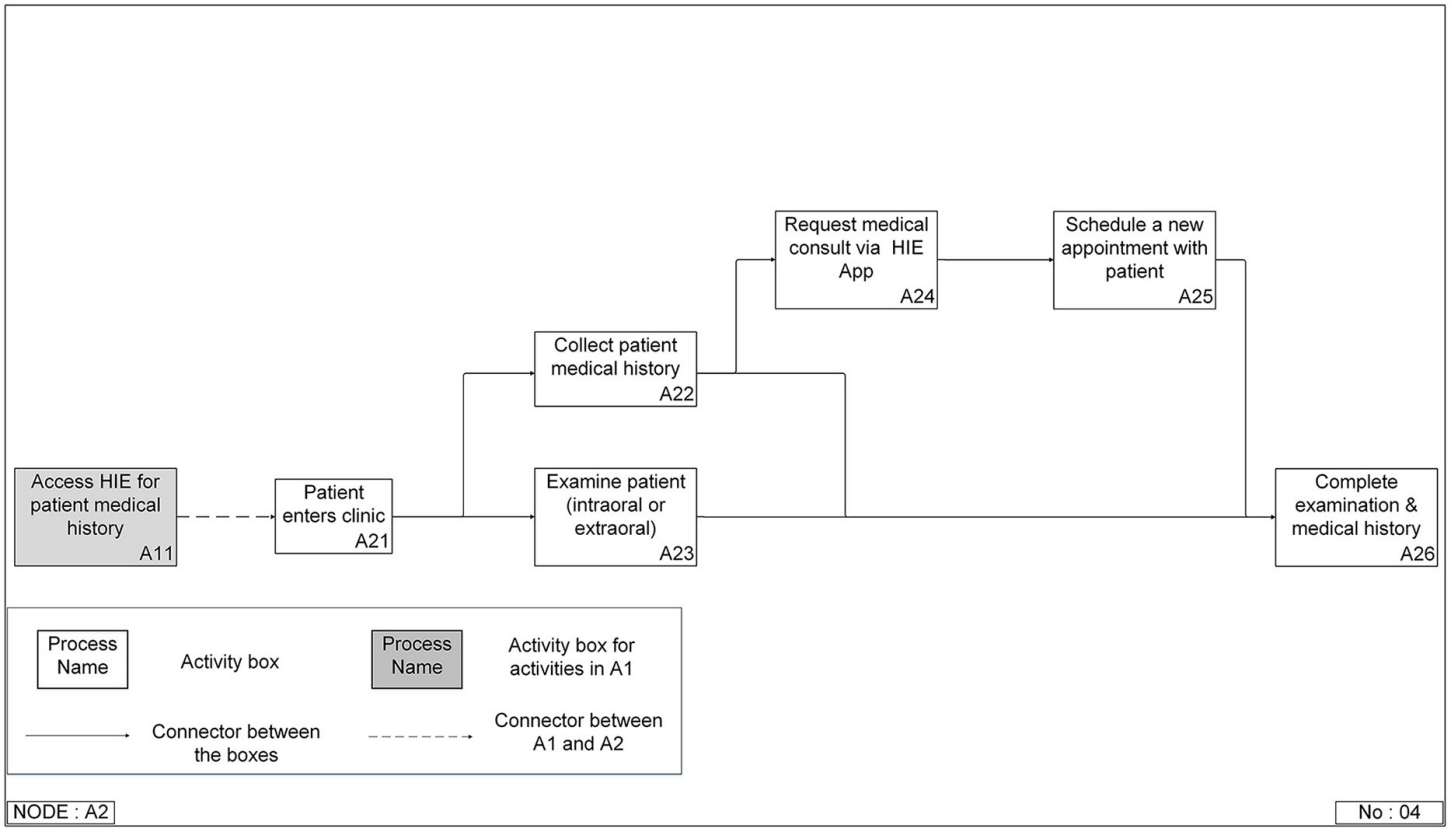
Integrated DEFinition Method Function (IDEF0) model for the health information exchange (HIE) approach that can connect standalone dental practices not affiliated with major healthcare organizations with community or regional HIEs to access their patient's medical history.

**Figure 11:**
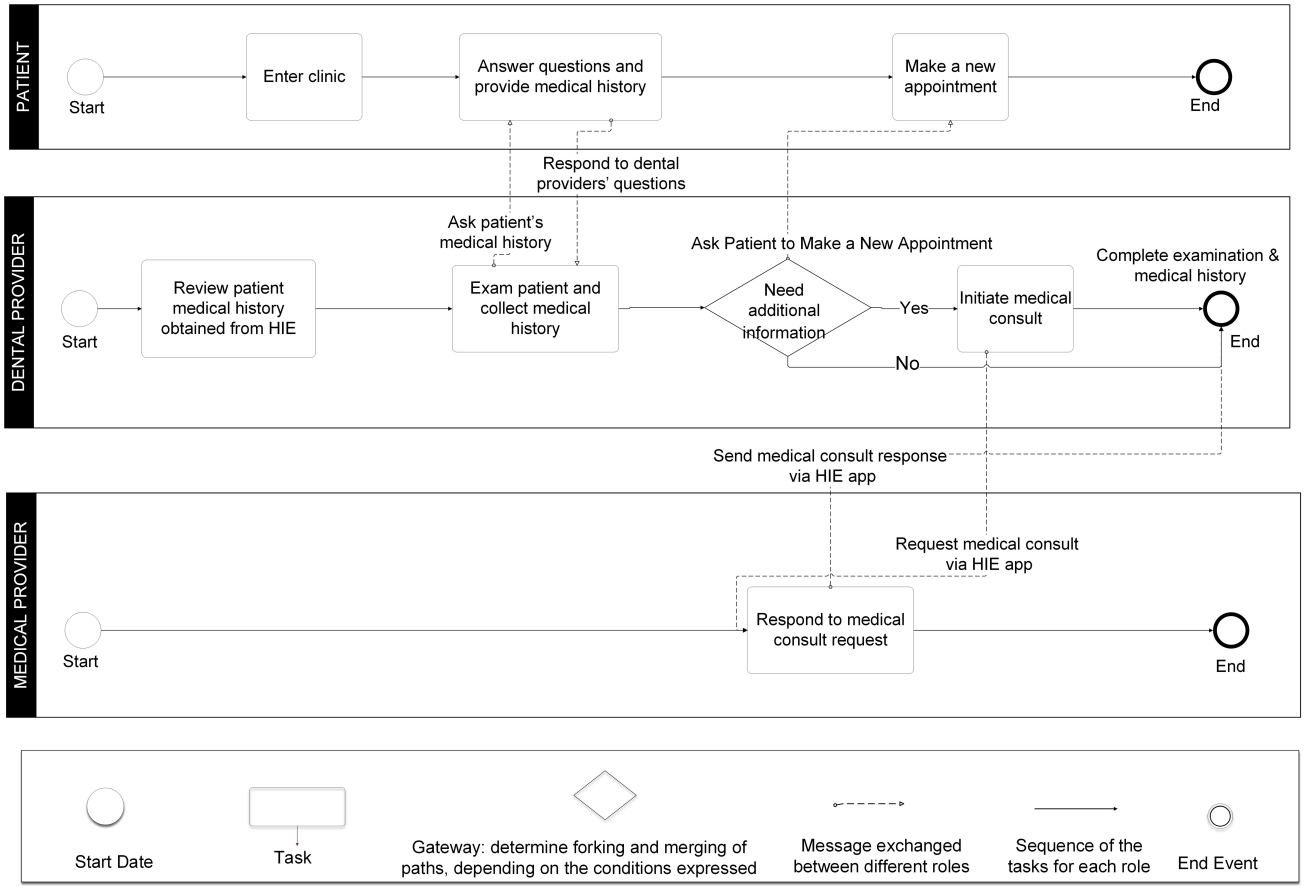
Business Process Modeling Notation (BPMN) model for the health information exchange (HIE) approach that can connect standalone dental practices not affiliated with major healthcare organizations with community or regional HIEs to access their patient's medical history.

The steps to access patient medical history through HIE and integrated EDR-EHR systems are similar (Figures 9, 10, 11). The major difference is the data source used in the two approaches. In the integrated EDR-EHR approach, the user has access to the patient medical history present in the EHR system of that healthcare organization. However, in the HIE approach, the user has access to the patient medical history present in the EHR systems of multiple healthcare organizations (including physician and specialty offices) that contribute data to the HIE. Thus, the HIE approach provides a more consolidated view of patient information than the integrated EDR-EHR, which includes information present in one system and one healthcare organization.

We also compared the current approaches and the HIE approach. The advantages and disadvantages of each approach were listed in Table 2.

**Table 2:** The strengths and weaknesses of the three approaches of dental clinicians accessing patient medical information identified through the modeling process.

**Patient-reported medical history with optional medical consults**	**Integrated electronic dental record (EDR)-electronic healthcare record (EHR)**	**Health information exchange (HIE)**
•Flexible	•Highest level of integration	•Support multiple systems integrations
•Suitable for complex questions or questions requiring explanation/interpretation	•Single system, single database, and information structure	•Large available datasets
•Longer time, may involve multiple requests	•Suitable for large healthcare enterprises and institutions	•Suitable for small and independent practices
•Low response rate, low-quality responses	•High IT involvement	•High information technology (IT) involvement
•No good way to trace	High cost	Low cost*
•Minimal IT involvement	•High system complexity	•Low system complexity*

* *Comparing to the integrated EDR-EHR system*.

## Discussion

Accessing up-to-date patient medical histories is essential for providing high-quality dental care. However, previously published works were focused on evaluating the quality of the collected patient medical histories, observing the benefits of the integrated EDR-EHR systems, and teaching dental students to communicate with medical providers and patients (66–69, 89). In the reviewed literature from 2013 to 2020, no studies have focused on modeling the information gathering process. In addition, only few studies exist on developing and implementing an information technology system to support the communications between dental and medical clinicians. We have modeled three different approaches for DCs to gather patient medical histories by using the UML use case diagram, the IDEF0 modeling method and the BPMN method in this study. The significant findings included (1) information technology tools that can help to improve the efficiency of the process; (2) a combination of information technology tools and medical consults could be the future approach; and (3) the HIE approach can provide benefits for reducing unnecessary medical consults and delay of care and for improving information quality but is more affordable for small independent practices. In the sections below, we discussed our findings and recommendations to improve the quality of the patient medical information available to the DCs and optimize the information gathering process.

### Medical Consults Will Not Be Replaced but Reduced

Medical consults will still be an essential function in the approaches using information technologies (both integrated EDR-EHR and HIE). It is the best approach for dental clinicians to get information on rare conditions and complex situations, including when dental clinicians need expert interpretations on the information. Information technology tools cannot, and we should not allow them, to be the only source for the management of all patient's medical information. Whether a complex integrated EDR-EHR system or an interface to retrieve data from an HIE is used, information technology tools should focus on the 80% of the most required interoperability needs (90). In clinical practices, there can be numerous different patient cases. The effort of exhausting all the possibilities will significantly increase the cost and the development time of the tools, leading to information overload. The clinicians can be buried in a lot of unnecessary data and be blocked from accessing the most needed ones. Future software tool design should focus only on the most needed information and leave the remaining ones to check with patients as currently performed or through medical consults. However, the medical consult process should be optimized to reduce unnecessary requests, improve the quality of the requested questions, and enhance the traceability for necessary requests.

### Future Studies Are Needed to Understand Dental Clinician's Information Needs

DCs' information needs are critical for future process optimization and software tool design. These can help to decide the size of the data and to choose the correct design and technology. The right set of information can dramatically improve the tool's usability and the efficiency of the process. If the clinicians can get the information they need directly, there will be fewer repeated steps. Clinicians' information needs have drawn attention in recent years, as reflected in prior studies on medical providers' information needs in an integrated medical-dental care system (57). However, the studies on DCs' needs are still rare and underdeveloped. Future studies should be two folded. One is to study the current medical consults including both the DC's requests and the medical provider's responses, to identify not only the most frequently requested types of information but also the gaps and inconsistencies between the requested and the responded information. The other one is to conduct survey studies and interviews/focus groups with DCs to refine and validate the types of information identified in the first study and collect DCs needs and preferences on how the information should be delivered and presented.

### The Inefficient and Cumbersome Process of Accessing Information and Poor Usability Hinder HIE Usage

Medical providers have been using HIE to retrieve patient's medical histories for more than a decade. Although HIE use shows improved healthcare benefits, variations exist in using HIE tools even with emergency departments which have logged higher HIE usage rates to access patient information (21, 87). This vast variation is due to the user's need to have multiple logins, workflow interruptions, and poor information display, thus hindering effective information retrieval and decision-making (87, 91). Efforts have been made by several state-wide HIEs to promote dentist's use of HIE to improve access to patient information (92, 93). However, a study that examined two New York FQHC dentist's use of the Rochester regional HIE demonstrated only 0.17% use of query based HIE during dental encounters (93). The current HIEs do not have specially designed services to meet DC's unique information needs and cannot support their information collection effectively and efficiently.

### Equal Access to New Technologies Requires Both Technical and Business Efforts and Public-Private Partnerships

To date, most dental clinicians are still working in private independent practices. The high financial and technical demands of a complex information system make it very difficult for small independent practices to adopt these new technologies (14, 20, 25). Efforts need to be made in both the technical and business fields to provide equal opportunities for clinicians to obtain easy and expedient access to the most needed information. This access will support clinicians in their efforts to provide high-quality dental care. From a technical point of view, developing easy-to-use and low-maintenance tools should be a high priority. From the business point of view, new business models should be established to reduce the financial burden on accessing information. Public-private partnership is another essential factor to improve the equality of accessing new technologies and providing dental care to all people. The National Institute of Health (NIH) published its Oral Health in America report in 2021, and the report has emphasized the importance of critical partnerships at all levels of the society to promote oral health and prevent disease (94). These partnerships may be established among various stakeholders at national, state, and community levels, including the federal agencies, state governments, academic institutions, healthcare organizations, patient advocacy organizations, and local communities.

### Limitations

The study modeled the user activities, the functions, and the processes of a new patient's first visit to a dental clinic. This modeling work is a valuable step in future development of information technology tools and further optimization of communications processes to support interprofessional collaboration during dental care. However, there are some limitations as it is our first effort to model this complex system. An expanded systematic review including gray literature and current applied models across geographies will be helpful to increase the generalizability of the models. We are also aware of the potential publication bias because small practices may not publish their work. This work is only the first step which help us to setup the framework and protocol on a much larger scale of study. We have planned surveys, key informant interviews, and focus group studies to invite DCs across US to provide their opinions and perceptions so we can further optimize the models and make them more comprehensive and accurate. In the current study, we only focused on modeling the activities, the functions, and the processes within the system. Other elements such as the data/information exchanging in the system, the interfaces between different subsystems, and the standards/rules applied to the system were not covered. The models can also be further broken down. The validation of the current models was conducted by domain experts and informaticians, future studies should expand the validation efforts with other stakeholders including patients, vendors of information technology tools, and healthcare organization managements to further refine the models.

## Conclusion

We successfully modeled the DC's current approaches of accessing patient medical history and designed an HIE approach that addressed the current approaches' weaknesses as well as leveraged their strengths. Organizational management and end-users can use this information to decide the optimum approach to integrate dental and medical care. The illustrated models are comprehensive and can also be adopted by EHR and EDR vendors to develop a connection between dental systems and HIEs.

## Data Availability Statement

The original contributions presented in the study are included in the article/supplementary material, further inquiries can be directed to the corresponding author.

## Author Contributions

SL and AR contributed to the creation of the models. AR, GF, and SL conducted the literature review. TT, TS, and EM were the domain exports who reviewed the models. SL, AR, and TT made significant contributions in the drafts of the manuscript. All authors contributed to manuscript revision, read, and approved the submitted version.

## Conflict of Interest

The authors declare that the research was conducted in the absence of any commercial or financial relationships that could be construed as a potential conflict of interest.

## Publisher's Note

All claims expressed in this article are solely those of the authors and do not necessarily represent those of their affiliated organizations, or those of the publisher, the editors and the reviewers. Any product that may be evaluated in this article, or claim that may be made by its manufacturer, is not guaranteed or endorsed by the publisher.
